# Style-Consistent Image Translation: A Novel Data Augmentation Paradigm to Improve Plant Disease Recognition

**DOI:** 10.3389/fpls.2021.773142

**Published:** 2022-02-07

**Authors:** Mingle Xu, Sook Yoon, Alvaro Fuentes, Jucheng Yang, Dong Sun Park

**Affiliations:** ^1^Department of Electronics Engineering, Jeonbuk National University, Jeonbuk, South Korea; ^2^Core Research Institute of Intelligent Robots, Jeonbuk National University, Jeonbuk, South Korea; ^3^Department of Computer Engineering, Mokpo National University, Jeonnam, South Korea; ^4^College of Artificial Intelligence, Tianjin University of Science and Technology, Tianjin, China

**Keywords:** tomato disease recognition, data augmentation, image translation, image classification, instance segmentation, image style

## Abstract

Deep learning shows its advantages and potentials in plant disease recognition and has witnessed a profound development in recent years. To obtain a competing performance with a deep learning algorithm, enough amount of annotated data is requested but in the natural world, scarce or imbalanced data are common, and annotated data is expensive or hard to collect. Data augmentation, aiming to create variations for training data, has shown its power for this issue. But there are still two challenges: creating more desirable variations for scarce and imbalanced data, and designing a data augmentation to ease object detection and instance segmentation. First, current algorithms made variations only inside one specific class, but more desirable variations can further promote performance. To address this issue, we propose a novel data augmentation paradigm that can adapt variations from one class to another. In the novel paradigm, an image in the source domain is translated into the target domain, while the variations unrelated to the domain are maintained. For example, an image with a healthy tomato leaf is translated into a powdery mildew image but the variations of the healthy leaf are maintained and transferred into the powdery mildew class, such as types of tomato leaf, sizes, and viewpoints. Second, current data augmentation is suitable to promote the image classification model but may not be appropriate to alleviate object detection and instance segmentation model, mainly because the necessary annotations can not be obtained. In this study, we leverage a prior mask as input to tell the area we are interested in and reuse the original annotations. In this way, our proposed algorithm can be utilized to do the three tasks simultaneously. Further, We collect 1,258 images of tomato leaves with 1,429 instance segmentation annotations as there is more than one instance in one single image, including five diseases and healthy leaves. Extensive experimental results on the collected images validate that our new data augmentation algorithm makes useful variations and contributes to improving performance for diverse deep learning-based methods.

## 1. Introduction

Food security has been raised to a high level in many countries partly because food distribution is not compatible with the distribution of the population in the world, and the number of laborers related is becoming less. While enough amount of food is required to feed our humans, many factors may harm plant growth and hence threaten the food supply. Controlling disease is one of the key challenges to keep plants healthy toward obtaining the expected yield. Artificial intelligence has recently witnessed a booming development with a decent disease recognition performance as intelligent machines deployed in farms can reduce workload. Deep learning, a core technique of artificial intelligence, has been successfully adopted to recognize diseases or abnormalities, such as tomato (Fuentes et al., 2017; Liu and Wang, 2020; Wang et al., 2021), banana (Lin et al., 2021), potato (Gao et al., 2021), corn and apple (Zhong and Zhao, 2020), and many other plants (Gao et al., 2020; Liu and Wang, 2021). Recent studies (Martineau et al., 2017; Liu and Wang, 2021; Saranya et al., 2021) show the advantages and potentialities of deep learning methods compared to other methods, such as handcraft feature, in recognizing plant diseases and related tasks.

To obtain a competing performance with a deep learning algorithm, enough amount of annotated data is requested but in the natural world, scarce or imbalanced data are common, and annotated data is expensive or hard to collect. For example, some diseases rarely appear or even never appear on one farm but the healthy plant is more common, which can not lead to a convincing disease recognition performance. To address this challenge, data augmentation is one of the most potential solutions and has been utilized in the agricultural field (Zhu et al., 2018; Nazki et al., 2020; Abbas et al., 2021). Data augmentation aims to generate more data with collected *training* data to improve the deep learning model's performance in *testing* data. Previous studies (Pawara et al., 2017; Douarre et al., 2019; Pinto Sampaio Gomes and Zheng, 2020; Liu and Wang, 2021; Saranya et al., 2021) have validated that data augmentation plays a significant role to improve the performance of deep learning in the agricultural area. In this study, we are interested in image-based recognition for tomato diseases, and hence data augmentation defaults with image-based.

**Figure 1:**
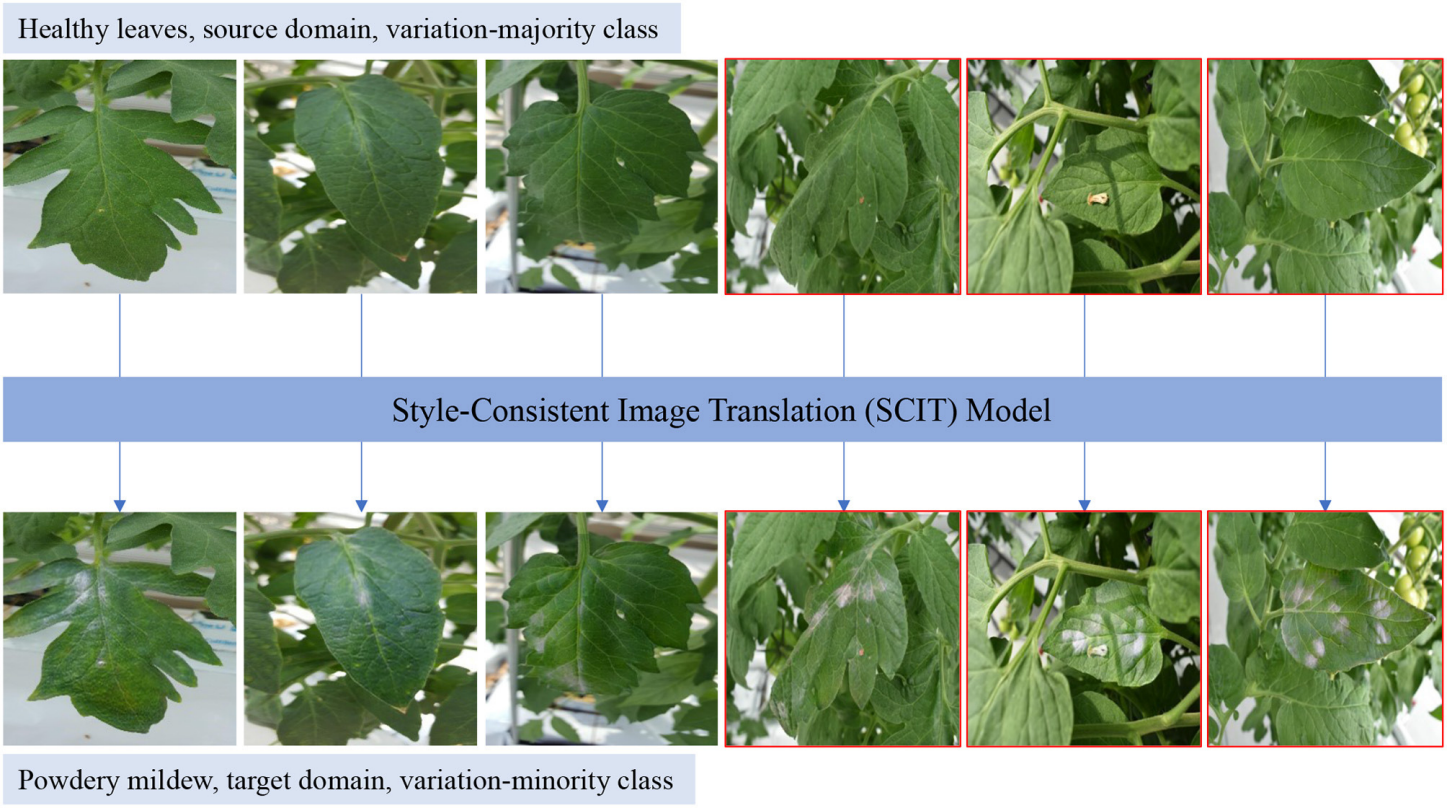
Data augmentation from healthy tomato leaves to powdery mildew leaves using the proposed style-consistent image translation (SCIT) model. Healthy leaves are in the source domain, belonging to the variation-majority class, while powdery mildew leaves are in the target domain, belonging to the variation-minority class. Ours SCIT model is leveraged to translate the images in the source domain into the target domain, which can take the variations from the variation-majority class to the variation-minority class. Healthy leaves include several variations, background, type of tomato, viewpoint, shape, illumination, size. SCIT model can *only* translate one given leaf in one image as shown right three images with red boundary, compared to other generative adversarial networks (GAN)-based data augmentation methods which translate all leaves in one image (Cap et al., 2020; Nazki et al., 2020).

Traditional data augmentation methods generate new augmented data within one specific class, *within-class data augmentation*, where the appearance of the image can be changed but the corresponding class remains, such as rotating or translating an image (Hu et al., 2020; Gorad and Kotrappa, 2021). In contrast, *cross-class data augmentation* methods can translate one image from the class to another class *via image translation* that aims to translate images from a source domain to a target domain, by which the variations are desired to be borrowed from one class to another class. For example, a healthy tomato image, source domain, can be translated into a powdery mildew image, target domain. In general, healthy tomato leaf images are easy to collect with large variations, such as background, viewpoint, size of the leaf, and type of tomato, as shown in Figure 1. As there is a high variation for the healthy tomato images, we refer the healthy to a *variation-majority class*. On the other hand, the class, hard to obtain images or enough variation, is referred to as a *variation-minority class*, such as some disease tomato leaves. To achieve the cross-class data augmentation, CycleGAN (Zhu et al., 2017), one of the state-of-the-art methods to do image translation, is plausible to be utilized. Based on CycleGAN, Nazki et al. (2020) proposed an activation reconstruction loss to improve the quality of the generated image. Except for low image quality, CycleGAN tends to change the undesired content such as background, and an attention module was proposed in LeafGAN (Cap et al., 2020) to detect the area that we are interested in. Although they achieved better results than the original CycleGAN, the following two new challenges are addressed in this study.

*First, how can we adapt the majority of variations from the source domain to the target domain?* Image translation is expected to keep the variations but there are no guarantees to keep the variations in current algorithms. To ease this issue, we propose a new paradigm to combine style consistency and image translation to maintain the variations during the image translation process, and hence, we call our algorithm style-consistent image translation (SCIT). Specifically, we follow the hypothesis that images can be factorized into two parts, *label-related* and *style-related* (Gonzalez-Garcia et al., 2018; Lee et al., 2018). While the label-related are the characteristics or patterns of specific classes such as healthy and powdery mildew, the style-related is independent of the labels, such as illumination, viewpoints, and background. In the translation process, we aim to translate the label-related but keep the style-related, which contributes to adapting the styles from the source domain to the target domain. In Figure 1, we can see that the selected healthy tomato leaves are translated into powdery mildew leaves by our method, but the styles in healthy tomato leaves are maintained, and the variations of powdery mildew domain are augmented from the healthy domain.

**Figure 2:**
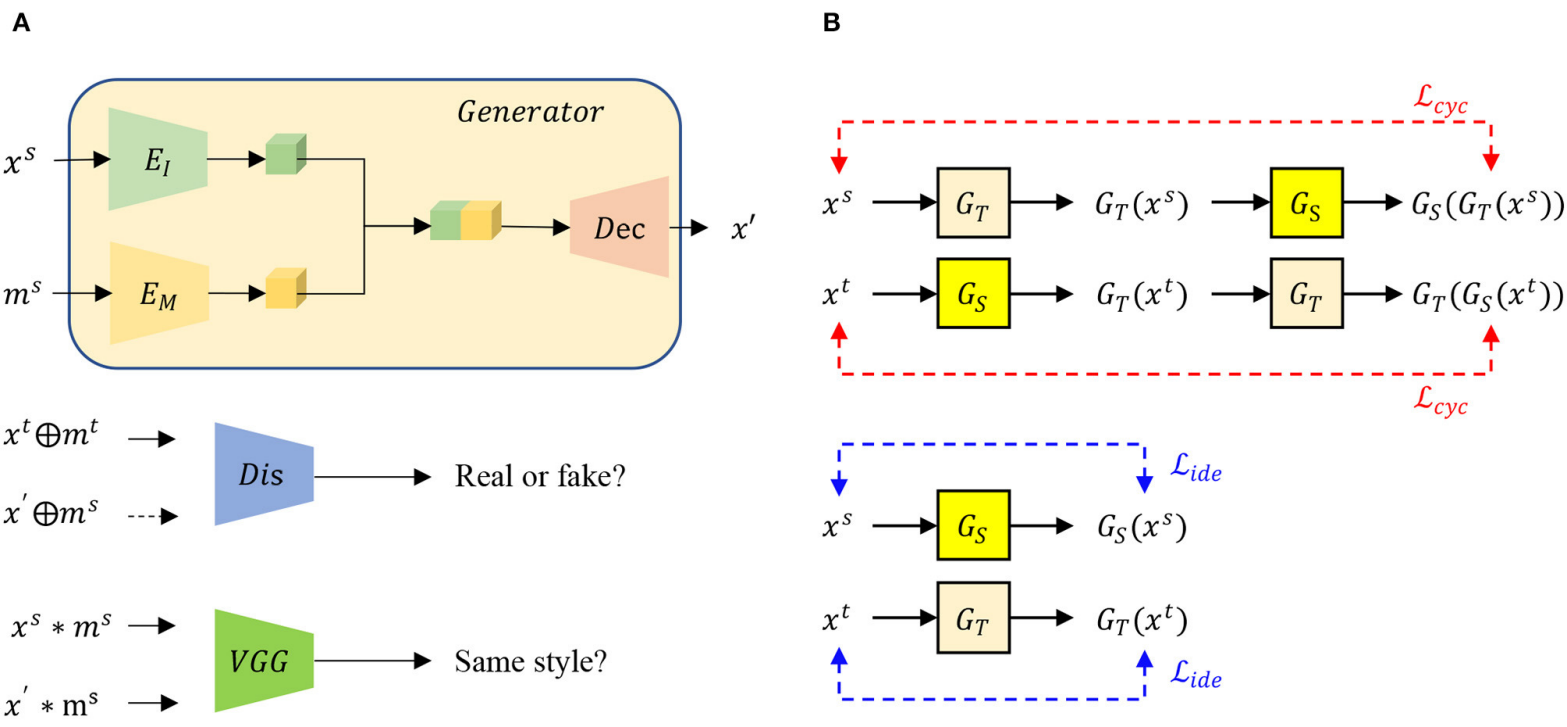
**(A)** Proposed SCIT model, the general structure of the proposed data augmentation model for image classification, object detection, and instance segmentation. *x*^*s*^ and *x*′ denote the source image and the generated image by our model. While *m*^*s*^ denotes instance segmentation mask aligning source image *x*^*s*^ and expecting to align *x*′, *m*^*t*^ is the instance segmentation mask of real images in the target domain. The generator consists of an image encoder *E*_*I*_, a mask encoder *E*_*M*_, and a decoder *Dec*. Discriminator *Dis* pushes the region of interest (ROI) of the generated image to have the same label with the ROI of the real image in the target domain, while the pre-trained VGG pushes ROIs to share the same style. **(B)** Flow chart to compute identity loss and cycle-consistent loss. *G*_*T*_ and *G*_*S*_ is the generator to produce images in the domain *T* and *S*, respectively. The masks to generate the image are omitted.

*Second, how can we use image translation as data augmentation to ease object detection and instance segmentation?* Traditional data augmentation and current image translation-based algorithms can be leveraged to improve the image classification but may not be appropriate to alleviate object detection and instance segmentation which are closer to our practical applications. The first reason is that object detection and instance segmentation require more annotations than image classification but current algorithms can not make those necessary annotations. Generally, we only need class information of images to train the image classification model, but the exact locations of each class are necessary to train the object detection algorithm, and both location and instance identity are required to train the instance segmentation model, **Figure 9** giving examples about the two tasks. Furthermore, classification is an image-level task but object detection and instance segmentation are at the instance level. Elusively, the input image undergoes a preprocessing to be one leaf or even part of one leaf to do image classification by which classification model is easier to be trained (Nazki et al., 2020). In contrast, preprocessing is not necessary for object detection and instance segmentation. In fact, the leaves are normally in different scales, as shown in the red boundary images in Figure 1. Therefore, we are requested to translate leaf instances separately in diverse scales instead of translating all leaves as other algorithms have been doing (Cap et al., 2020; Nazki et al., 2020). To address the two issues, we employ a mask as prior knowledge in order to split an image into the region of interest (ROI) and background (BG). We aim to translate its ROI part but reuse its BG part, by which the original annotations can be reused for the produced images. Further, we design a new framework based on CycleGAN (Zhu et al., 2017). First of all, a mask encoder is designed to be incorporated with the image encoder, as shown in Figure 2, by which our generator knows where is interested. Although a similar idea appears in RBGAN (Xu et al., 2021), we aim to translate part of the image yet keep the other part and maintain the style during the image translation but RBGAN aims to perform instance-level image translation with a decent translated instance boundary. Besides, our discriminator absorbs both real or fake images and corresponding masks and is pushed to know where is the translated area and whether the area is real or fake. Therefore, with the new generator and discriminator along with the input mask, our algorithm can translate given leaves in diverse scales with the reused annotations, which contributes to ease object detection and instance segmentation as a data augmentation method.

To summarize, our contributions are as follows:

1. We propose a data augmentation paradigm, SCIT, which can increase the data variations for the variation-minority class by leveraging the images from the variation-majority class.
2. We propose a framework to perform image translation in diverse scales with necessary annotations as output to ease object detection and instance segmentation, which is out of current data augmentation methods.
3. Taking tomato as an example, we perform extensive experiments on three tasks, image classification, object detection, and instance segmentation. The experimental results suggest that our proposed algorithm improves the performances for diverse deep learning-based methods and outperforms the state-of-the-art data augmentation methods.

The remainder of this study is organized as follows. Related studies and our basic idea are introduced in the preliminary section. The proposed method to do data augmentation is instantiated in Section 3, including the framework and loss function. In the experiments section, we show the details about our dataset, implementation to train and test our model, ablation study to understand our algorithm, comparison to other methods in three tasks. Finally, we conclude our studies and future study in the last section.

## 2. Preliminary

Data augmentation based on the image can be categorized into two main parts, basic image manipulations and deep learning-based algorithms. In this section, we try to highlight the difference between other methods and our method to achieve data augmentation.

**Image manipulations**. Image manipulations make use of image processing methods, such as pixel-wise conversion and geometrical transformations. Formally, let *x*^*s*^ and *x*^*a*^ denote the source image and the augmented image. Similarly, *y*^*s*^ and *y*^*a*^ are corresponding labels. The formulation of basic image manipulation-based data augmentation refers to Equation 1.


(1) $$\left\{ \begin{array}{l} x^{a}=f_{m}(x^{s}),\\ y^{a}=y^{s}, \end{array}\right.$$

where *f*_*m*_ is one of the basic image manipulation functions. The equation suggests that the label of the input image is not changed when the input image undergoes manipulations. The retainment degree of the label is called safety of data augmentation because manipulations can not always keep the label (Shorten and Khoshgoftaar, 2019). For example, after adding too much random noise, some images could not be recognized as before. With these image manipulations, prior works achieved better performance in the missions with small datasets (Hu et al., 2020; Gorad and Kotrappa, 2021). Besides, these basic image manipulation can be adapted from one image to more than one image (Dwibedi et al., 2017). Kuznichov et al. (2019) copied the leaves from different images to form a new augmented image to promote leaf segmentation and counting. Similarly, Gao et al. (2020) produced a synthetic image by combining specific objects from different images, in which two different classes can appear in a single image. Their experiments validated that the performance can be also improved by their methods. In this study, we aim to do data augmentation from another viewpoint by using one class to augment another class, hoping that more variations can be produced.

**Deep learning-based algorithms**. Different from image manipulations, deep learning-based algorithms employ deep neural networks to generate new images. According to the condition to generate new images, deep learning-based algorithms can be split into label-condition and image-condition. Label-condition algorithms generate images from given labels by using generative adversarial networks (GANs) (Valerio Giuffrida et al., 2017; Pandian et al., 2019; Bi and Hu, 2020; Abbas et al., 2021). In contrast, image-condition algorithms produce images from given images. Style transfer is one of the possible methods (Li et al., 2017; Huang et al., 2021; Shen et al., 2021). Mathematically, it can be formalized as Equation 2.


(2) $$\left\{ \begin{array}{l} x^{a}=f_{st}(x^{s}),\\ y^{a}=y^{s}, \end{array}\right.$$

where *f*_*st*_ is the style transfer function. Because of the function, style transfer algorithms can introduce more variations into the dataset. For instance, images captured in sunny style can be transferred into night style (Shen et al., 2021), which is beyond the basic image manipulations. However, the labels of the transferred images are considered as same as the source, which is the same as the basic image manipulations. Besides, the style transferring-based algorithms try to maintain the content of the image. Take the tomato leaves as an example, it aims to maintain the size, shape of leaves, viewpoints and, hence, making more variations about them is still challenging.

To deal with the challenge mentioned above, we propose a novel paradigm to achieve data augmentation, following the disentangled idea that an image can be factorized into two factors: style-related and label-related (Gonzalez-Garcia et al., 2018; Lee et al., 2018). By using image translation, label-related factors of an image in the source domain can be translated into images in the target domain. Simultaneously, the style-related factors of the source image are desired to be kept in the translated image. In the style-consistent image translation, the variations of the style in the source domain are borrowed into the target domain. Mathematically, this kind of data augmentation algorithm can be formulated as Equation 3.


(3) $$\left\{ \begin{array}{l} x^{a}=f_{it}(x^{s}),\\ y^{a}\neq y^{s},\\ g(x^{a})=g(x^{s}), \end{array}\right.$$

where *f*_*it*_ symbolizes image translation function. *y*^*a*^ and *y*^*s*^ denote the label corresponding to image *x*^*s*^ and *x*^*a*^, respectively. The function *g* symbolizes the style extracting function.

We argue that this kind of data augmentation eases practical applications. When it is hard to collect the data or the collected data suffer from a lack of variations in one domain but easier to collect data in another domain, we can use this data augmentation to leverage the variations in the easier domain to promote the variations in the harder domain. For example, images of healthy tomato leaves can be easily collected from farms but images with specific diseases of abnormalities like powdery mildew could not be collected easily, mainly because farmers must do necessary measures to prevent before their appearance or make a fast remedy after their appearance to reduce financial loss. We can augment data for powdery mildew effectively by using image translation from healthy domain to powdery mildew domain. Besides, we emphasize that the proposed data augmentation method can be employed with any kind of image translation model *f*_*it*_ and domain-invariant function *g*.

Moreover, we also notice that data augmentation for image classification attracts much more attention than for object detection or instance segmentation. A label is globally assigned to a whole image for the image classification task but a label is locally assigned to a region of an image for object detection or instance segmentation task. Therefore, one of the challenges for object detection and instance segmentation is mainly that dealing with a bounding box or instance mask for a local region is required. To address this issue, we further spatially split one image into two parts, region of interest (ROI) *x*_*roi*_ and background *x*_*bg*_. Formally, input image *x* is split into *x*_*roi*_ and *x*_*bg*_ according to the binary instance segmentation mask from source domain *m*^*s*^. Then our proposed generative adversarial network takes the *x*^*s*^ and *m*^*s*^ as input and aims to translate the *x*_*roi*_ into the target domain. Simultaneously, we reuse the background of the source image since the translation model is not interested in the background. In this way, the augmented image *x*^*a*^ shares the same bounding box or instance segmentation with the source image *x*^*s*^, but the label is changed. Formally, the SCIT for object detection and instance segmentation can be formalized as Equation 4.


(4) $$\left\{ \begin{array}{l} x^{a}=m^{s}*f_{it}(x^{s})+(1-m^{s})*x^{s},\\ y_{roi}^{a}\neq y_{roi}^{s},\\ g(x^{a})=g(x^{s}), \end{array}\right.$$

where $y_{roi}^{a}$ and $y_{roi}^{s}$ denote the label corresponding to the ROI of *x*^*a*^ and *x*^*s*^. We found this kind of method suitable for image classification.

## 3. Style-Consistent Image Translation

In this section, we instantiate *f*_*it*_ and *g* as a GAN model and VGG network and deploy them to do image classification (Simonyan and Zisserman, 2014), object detection, and instance segmentation for tomato leaves. Specifically, an updated CycleGAN (Zhu et al., 2017) is leveraged in our experiments to translate images from the source domain to the target domain. To keep the style consistent, VGG loss is employed (Huang and Belongie, 2017; Li et al., 2017). But we emphasize that other kinds of image translation methods and style losses are possible and encouraged. Since our method aims to keep the style consistent when doing image translation, it is called SCIT.

### 3.1. Framework

Style-consistent image translation consists of three parts functionally, as shown in Figure 2A. The Generator, *G* for short, is expected to translate the image, while the discriminator *Dis* is assumed to push the translated image similar to the real image in the target domain, and a pre-trained VGG19 is utilized to extract the style, class-unrelated characters.

The generator *G* absorbs source image *x*^*s*^ and instance segmentation mask from source image *m*^*s*^ as input, in which two specific encoders, *E*_*I*_ and *E*_*M*_, are leveraged to extract features from image and mask, respectively. The outputs of the two encoders are concatenated, followed by a decoder to produce an output image *x*′. Formally, the generating process can be formalized as Equation 5.


(5) $$\begin{array}{r} x^{\prime }=G\left(x^{s},m^{s}\right)=Dec\left(E_{I}\left(x^{s}\right)⊕E_{M}\left(m^{s}\right)\right), \end{array}$$

where ⊕ denotes concatenation channel-wise. The instance segmentation mask *m*^*s*^ is used to let the generator know where to put attention. Similarly, our discriminator takes in real image *x*^*t*^ in the target domain or generated image *x*′, along with its instance mask *m*^*s*^, to recognize whether it is fake or real.

In the inference time, the image *x*^*a*^ to be used as augmented data is a fusion of the source image *x*^*s*^ and the generated image *x*′. Equation 6 shows the inference process to get augmented data. Intuitively, the augmented image has the same background as the source image but has the same foreground as the translated image.


(6) $$\begin{array}{r} x^{a}=m^{s}*Dec\left(E_{I}\left(x^{s}\right)⊕E_{M}\left(m^{s}\right)\right)+\left(1-m^{s}\right)*x^{s}. \end{array}$$

### 3.2. Loss Functions

The loss functions employed to train our SCIT model are explained in this subsection. To push the generated image toward the real image in the target domain, GAN loss is used as shown in Equation 7.


(7) $$\begin{array}{r} L_{GAN}=𝔼||Dis\left(x^{t}⊕m^{t}\right)-1|{|}_{2}+𝔼||Dis\left(G\left(x^{s},m^{s}\right)⊕m^{s}\right)|{|}_{2}. \end{array}$$

Except for being like a real image in the target domain, the generated image *x*′ is hypothesized to have the same style as the source image *x*^*s*^, which is realized by a pre-trained VGG network, as Equation 8.


(8) $$\begin{array}{r} L_{sty}=𝔼\overset{n}{\underset{i}{\sum }}k\left(\phi_{i}\left(x^{s}*m^{s}\right),\phi_{i}\left(x^{\prime }*m^{s}\right)\right), \end{array}$$

where *k* denotes a kernel function, and ϕ is an image feature extractor. A linear kernel function is employed in this study, as it gives a decent performance and requires less computation (Li et al., 2017). We apply a pretrained VGG-19 network as the feature extractor without finetuning. Thus, ϕ_*i*_ means a layer in VGG-19, which is similar to the literature. In our experiments, we use *relu*1_1, *relu*2_1, *relu*3_1, *relu*4_1, and *relu*5_1 layers with equal weights and, thus, *n* = 5. Intuitively, the style loss pushes the two images to share the same feature distribution. Specifically, the linear kernel function-based loss encourages them to have the same sample mean in feature distribution space. We refer to Li et al. (2017) to check the detail about the style loss and its related kernel functions. The deep learning-based style loss with a linear kernel function is adopted in our experiment, but other methods, such as different kernel functions and new style loss are theoretically possible.

To ease the training of generator model *G*, identity loss and cycle-consistency loss are leveraged in our experiments, as shown in Figure 2B (Zhu et al., 2017). To describe clearly the cycle-consistency loss, we use subscript with *S* and *T* to denote the source domain and target domain. For instance, *G*_*S*_ means the generator which aims to translate an image into the source domain while *G*_*T*_ denotes the generator which aims to translate an image into the target domain. Identity loss, defined in Equation 9, comes from that when one instance in the target domain is given to generator *G*_*T*_ as input, the generator needs to output the same as its input without any change. Equation 10 shows the way to calculate the cycle-consistent loss. When we translate an image in the source domain into the target domain and translate the result back into the source domain, we want to get the same result as the original input.


(9) $$\begin{array}{r} L_{ide}=𝔼||G_{S}\left(x^{t},m^{t}\right)-x^{t}|{|}_{1}+𝔼||G_{T}\left(x^{s},m^{s}\right)-x^{s}|{|}_{1}. \end{array}$$

(10) $$\begin{array}{r} L_{cyc}=𝔼||G_{S}\left(G_{T}\left(x^{s},m^{s}\right),m^{s}\right)-x^{s}|{|}_{1}+𝔼||G_{T}\left(G_{S}\left(x^{t},m^{t}\right),m^{t}\right)-x^{t}|{|}_{1}. \end{array}$$

In the end, our model is trained with four loss functions described before. Mathematically, Equation 11 shows the sum of the four losses, where λ_*_ balances each loss.


(11) $$\begin{array}{r} L_{full}=L_{GAN}+\lambda_{sty}L_{sty}+\lambda_{ide}L_{ide}+\lambda_{cyc}L_{cyc}. \end{array}$$

## 4. Experiments

### 4.1. Dataset and Implementation Details

**Figure 3:**
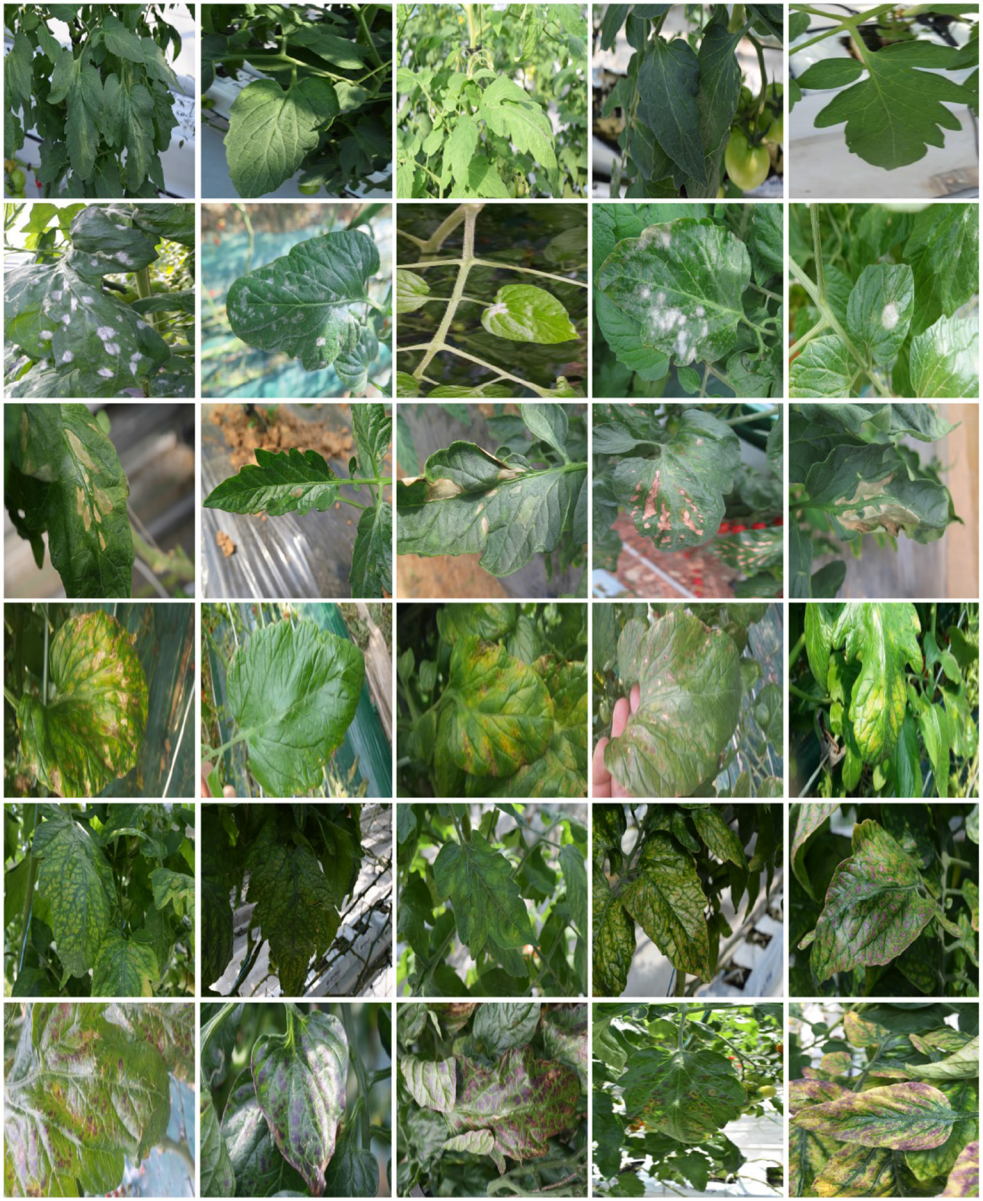
Original tomato leaf dataset. From the first to the last row are Healthy, Powdery (powdery mildew), Canker, LMold (leaf mold), ToCV, MagDef (magnesium deficiency). We collect the dataset from different farms at different times. The variations among the dataset include background, type of tomato, the severity of disease, illumination condition, the distance between the camera and interesting leaf, viewpoint to take the picture.

**Figure 4:**
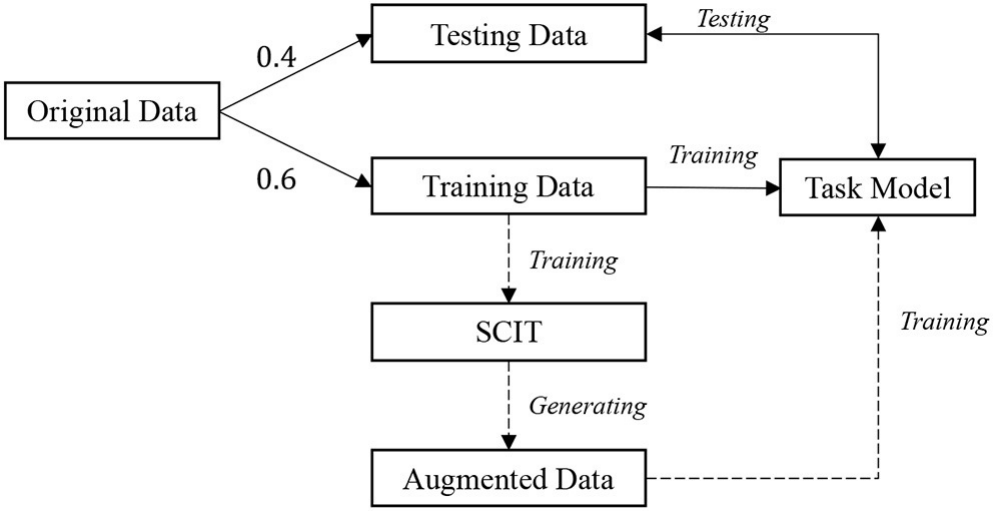
Data utilization process. The originally collected data are split into testing and training data, 0.4 and 0.6, respectively. The training data is firstly adopted to train our SCIT model that then generates the augmented data. Next, the training data along with the augmented data are leveraged to train the task model (image classification, object detection, or instance segmentation). The dotted lines suggest the data augmentation process. The SCIT model is one of our main contributions, which introduces new variations for the original data and, thus, encourages the task model to have better performance on the testing data.

**Dataset**. We aim to recognize tomato diseases among different farms and collect data in real farms with many variations, such as the type of tomato, the distance between the camera and tomato leaf, weather, and illumination. Figure 3 gives examples of the collected images. A total of 1,258 images of tomato leaves are collected, called original data, which covers five types of disease and healthy leaves. Table 1 displays the number of images for each class. As shown in Figure 4, the original data are first split into 40% testing and 60% training data, respectively. We adopt the training data to train data augmentation models and utilize all healthy leaves images as testing to get the augmented data for the other five diseases. Table 1 shows the number of augmented data for each class. Since we are not interested in healthy leaves, we do not do data augmentation for the healthy class. A total of 314 generated images and 358 instances are generated for each disease class. Finally, the augmented data and the training data are leveraged to train task models (classification, object detection, and instance segmentation). While object detection and instance segmentation can give more than one label or one instance to one image, image classification requires one holistic label for one image. To ease image classification, we crop the original image to get a single leaf in one image.

**Table 1:** Dataset used in the experiment.

	**Original data**						**Augmented data**	
	**All**		**Testing data**		**Training data**		
**Type**	**Images**	**Masks**	**Images**	**Masks**	**Images**	**Masks**	**Images**	**Masks**
Healthy	314	358	0	0	0	0	0	0
Powdery	170	185	71	73	106	112	314	358
Canker	298	309	119	122	185	187	314	358
Leaf mold	166	198	74	78	105	120	314	358
ToCV	172	227	81	89	115	138	314	358
Mag Def	138	152	57	59	86	93	314	358
All	999	1,071	402	421	597	650	1,570	1,790

*It includes healthy tomato leaves and five kinds of diseases of abnormalities. To do the tasks (image classification, object detection, and instance segmentation), the original data are split into two parts according to the number of masks, 60% training and 40% as testing. The healthy images are only used to do image translation*.

**Implementation details**. The original training dataset is employed to train our data augmentation model. Meanwhile, basic image manipulation is adopted to enlarge the original data since the collected data are not enough to train the SCIT model. Specifically, we use three times random brightening or darkening, and three times random cropping. Hence, the training data are enlarged to six times in total. During the training process of our data augmentation model, random flip in horizontal and vertical are employed and every image is resized to 256 in both height and width. Each type of disease employs one specific SCIT model.

We use Adam optimizer to train our model for 100 epochs with a learning rate of 0.0002. The batch size is set as 6 with three TITAN V GPUs (12 GB memory). After training, the trained models are adopted to generate disease images, which are later taken as augmented data to train task models. Every training process for each class and λ_*sty*_ roughly spends 5 h. Therefore, all translation models require about 26 days (5 translation models for each class and 5 λ_*sty*_ settings, each setting is executed 5 times.)

**Architectures**. The proposed SCIT model consists of three sub-models. First, the generator consists of an image encoder, mask encoder, and decoder. The image encoder, aiming to extract necessary information from the input images, leverages several stacks of convolution-ReLU-BatchNorm layers and nine residual blocks, while the mask encoder only adopts the same number of stacks of convolution-ReLU-BatchNorm layers without residual blocks since the mask is much simpler than images. In contrast, the decoder, aiming to produce bigger size images from the smaller size of the feature map, employs several stacks of deconvolution-ReLU-BatchNorm. Second, the discriminator also applies several stacks of convolution-LeakyReLU-BatchNorm. The details of our generator and discriminator are referred to in Table 2 and our codes will be public soon^1^. In terms of the style computation model, a pretrained VGG19 model^2^ is leveraged.

**Table 2:** The architecture details adopted in our algorithm.

**Network**	**Input size**	**Operation**	**Normalization**	**Active function**
*E*_*I*_	(256, 256, 3)	Conv7-C64-S1-P3	InstNorm	ReLU
	(256, 256, 64)	Conv3-C128-S2-P1	InstNorm	ReLU
	(128, 128, 128)	Conv3-C256-S2-P1	InstNorm	ReLU
	(64, 64, 256)	Residual block * 9		
*E*_*M*_	(256, 256, 3)	Conv7-C64-S1-P3	InstNorm	ReLU
	(256, 256, 64)	Conv3-C128-S2-P1	InstNorm	ReLU
	(128, 128, 128)	Conv3-C256-S2-P1	InstNorm	ReLU
*Dec*	(64, 64, 512)	Conv1-C256-S1-P1	InstNorm	ReLU
	(64, 64, 256)	DeConv3-C128-S2-P1	InstNorm	ReLU
	(128, 128, 128)	DeConv3-C64-S2-P1	InstNorm	ReLU
	(256, 256, 64)	Conv7-C3-S1-P0	InstNorm	Tanh
*Dis*	(256, 256, 3)	Conv4-C64-S2-P1	InstNorm	LeakyReLU
	(128, 128, 64)	Conv4-C128-S2-P1	InstNorm	LeakyReLU
	(64, 64, 128)	Conv4-C256-S2-P1	InstNorm	LeakyReLU
	(32, 32, 256)	Conv4-C512-S1-P1	InstNorm	LeakyReLU
	(31, 31, 256)	Conv4-C1-S1-P1	InstNorm	Sigmoid

*The input size is height, width, and channel. In the operation, Convk is a convolution layer with kernel size as k, DeConv is the deconvolution layer. Ck, Sk, and Pk denote the number of channels, stride, and padding, respectively. Instance normalization (InstNorm) is used. We utilize nine residual blocks (He et al., 2016) to extract necessary information from the image while no residual block is used in the mask encoder as the mask is much simpler than the image*.

### 4.2. Ablation Study

**FIDs and Visualization**. In this subsection, we analyze the impact of the style-consistent loss by changing the value of λ_*sty*_ in Equation 11. We use Fréchet inception distance (FID) (Heusel et al., 2017) to show its impact, one of the popular methods to access the quality of the generated images by computing the distance between two images distributions, real images, and the translated images. In general, the lower the FID value, the closer distance between the distributions. We point out that FID is not suitable to access the generated images by our SCIT model since we assume that the real images in the target domain are not available. But the FID can be used to show the tendency between our generated images and all available data that we have when λ_*sty*_ changes.

To compute the FID, all original images including training and testing images are leveraged as real images while the generated images are taken as fake images. We borrow code^3^ to compute FID. Table 3 shows the FID values for each class, λ_*sty*_ ranging from 0 to 4. From the table, we observe that FID tends to be larger as λ_*sty*_ ranges from 0.5 to 4, which proves that the generated images are farther from the real images when we try to keep the style in the image translation process. Moreover, the performance of λ_*sty*_ = 0 differs in different classes. It shows lower FIDs in LMold and MagDef but higher FIDs in translated powdery mildew.

**Table 3:** The impact of λ_*sty*_ on FIDs. For each λ_*sty*_, we execute five times and report the mean and SD.

	**λ_*sty*_ = 0**	**λ_*sty*_ = 0.5**	**λ_*sty*_ = 1**	**λ_*sty*_ = 2**	**λ_*sty*_ = 4**
Powdery	70.3 ± 1.35	60.2 ± 1.41	63.0 ± 1.40	65.7 ± 0.60	67.3 ± 0.84
Canker	97.5 ± 2.36	93.4 ± 2.90	93.5 ± 1.73	98.0 ± 2.81	99.3 ± 1.93
LMold	97.9 ± 3.09	98.2 ± 2.97	100.8 ± 2.34	103.1 ± 3.32	112.6 ± 5.14
ToCV	79.6 ± 5.95	72.7 ± 6.68	75.7 ± 5.35	86.6 ± 3.52	93.0 ± 2.14
MagDef	83.2 ± 4.10	83.1 ± 1.71	85.1 ± 1.77	90.2 ± 2.83	89.5 ± 3.12

**Figure 5:**
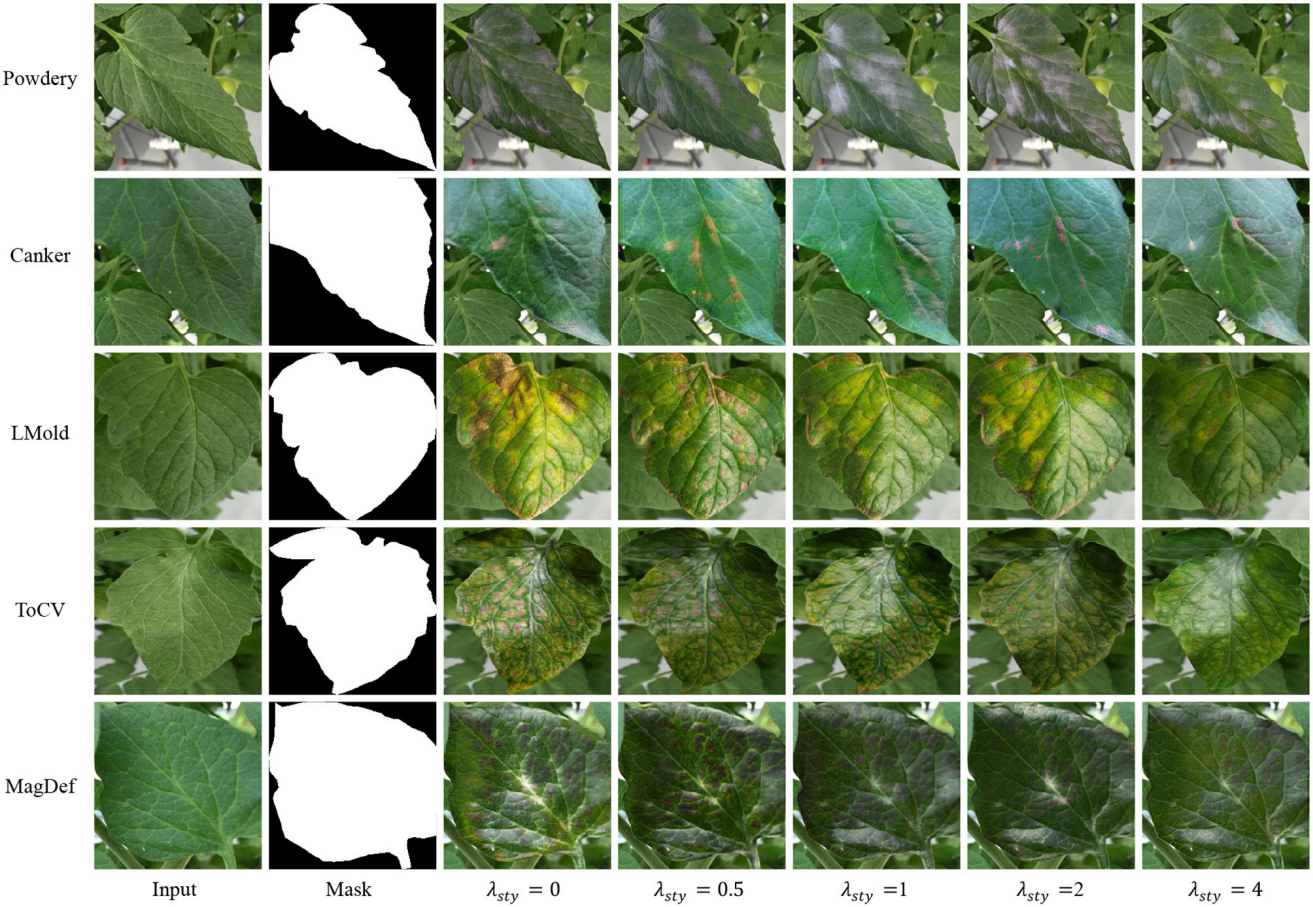
Some generated images for image translation with different λ_*sty*_.

Figure 5 shows the generated image with different λ_*sty*_. The visual comparisons in the figure comply with the FID values in Table 3. First, the generated images with λ_*sty*_ = 0 are clear to show the corresponding class, but the style is far from the input images. Further, the style is better to be maintained when λ_*sty*_ becomes larger while the abnormal severity tends to be less. We argue that the variation of the severity contributes to improving task performance. As the collected data are limited to variations, the FID in big λ_*sty*_ tends to be worst for some classes, such as in Lmold and MagDef. But when the variations in the collected data are bigger, such as powdery mildew, our SCIT model tends to be better.

**Image classification**. Except for FID and visualization, we conduct the ablation study on the image classification task. To compare the accuracy in image classification, three categories of classification models are utilized for our tomato leaves. VGG, ResNet, and DenseNet are often deployed in applications with big scale datasets, while MobileNet and ShuffleNet aim to save computations for mobile devices. MNASNet is designed to find the optimal model setting. As our dataset is not big, smaller architecture is the default for all models. On the other hand, since our main objective is data augmentation, we use an open code^4^ to produce it. For each model, we execute five times independently for each augmented data. All models are trained for 400 epochs and the best performance is recorded. The initial learning rate is set as 0.02 and decreased to 0.01 and 0.005 at epoch 50 and 200 respectively. SGD is the optimizer with 0.9 as the momentum and batch size is 64 using one GPU. Table 4 displays the comparison results. The table shows that the performance varies with different λ_*sty*_, λ_*sty*_ = 2 showing its superiority to other values except in MNASNet, and controlling the style tends to be better than without controlling the style, such as λ_*sty*_ = 0 resulting in less accuracy than other values of λ_*sty*_ with model ResNet, DenseNet, MobileNet, and ShuffleNet. It validates that our model, style controlling, is reliable to dedicate the classification in the applications with a small dataset. Moreover, the classification accuracy changes with different models. We argue that it is related to the model itself. As ResNet has fewer parameters and more powerful architecture than VGG, the performances in ResNet are better than VGG. DenseNet, an advanced version of ResNet, also obtains decent results. While MobileNet receives competing results, MNASNet behaves much lower than other methods. We guess that the optimal model setting of MNASNet learned from other datasets is not suitable for our tomato dataset.

**Table 4:** Ablation study of λ_*sty*_ in image classification.

	**VGG11**	**ResNet18**	**DenseNet121**	**MobileNet v2**	**ShuffleNet v2**	**MNASNet**
λ_*sty*_ = 0	89.04 ± 2.12	92.68 ± 0.63	96.01 ± 0.32	94.14 ± 0.23	79.56 ± 0.42	67.65 ± 7.01
λ_*sty*_ = 0.5	88.03 ± 3.75	94.24 ± 0.55	96.25 ± 0.46	95.56 ± 0.32	80.37 ± 0.19	65.08 ± 6.65
λ_*sty*_ = 1	93.82 ± 2.34	94.10 ± 0.64	96.24 ± 0.48	96.20 ± 0.28	86.46 ± 0.69	73.11 ± 4.52
λ_*sty*_ = 2	94.36 ± 2.63	94.61 ± 0.38	96.48 ± 0.44	96.28 ± 0.32	87.44 ± 0.69	69.45 ± 7.48
λ_*sty*_ = 4	86.17 ± 3.12	93.96 ± 0.76	95.32 ± 0.31	95.57 ± 0.49	82.35 ± 0.79	64.52 ± 6.79

*The red font shows the best accuracy for each classification model*.

**Figure 6:**
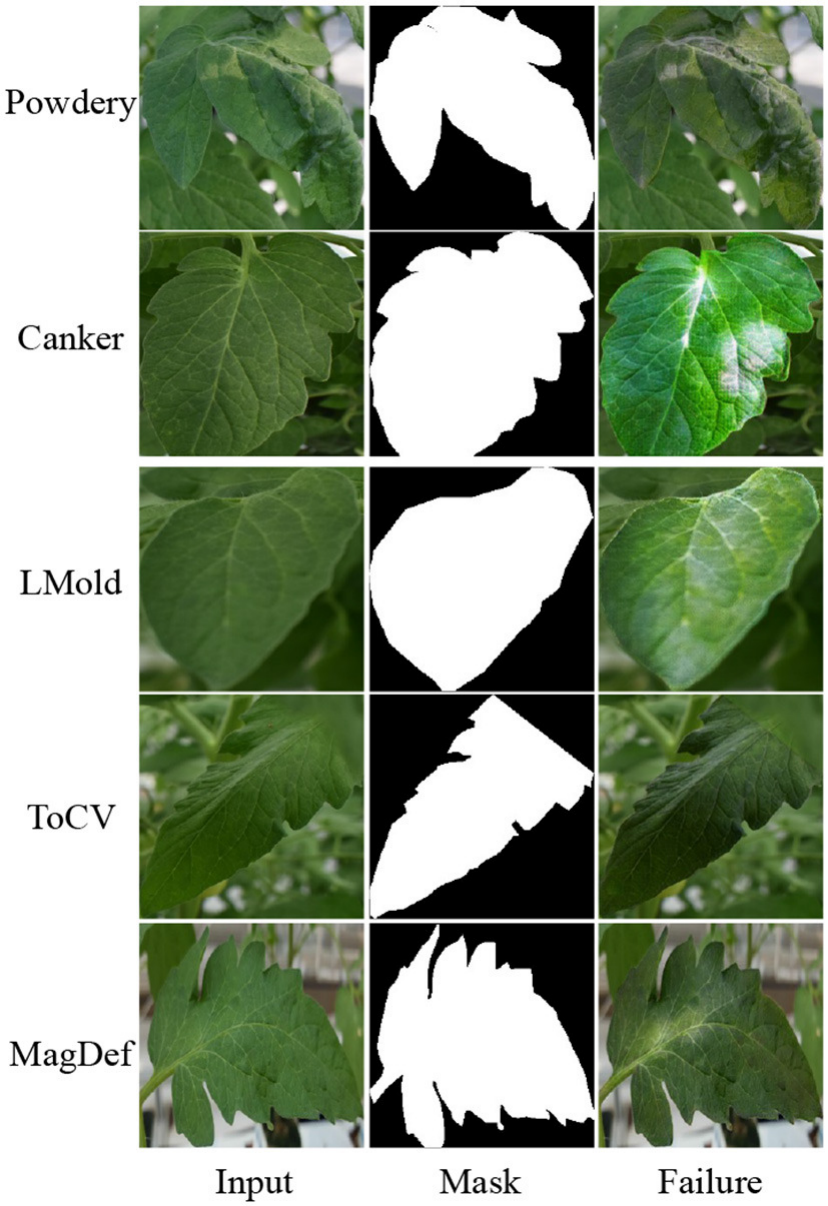
Some translation failure examples from our algorithm.

**Verification of SCIT**. As shown in Table 5, performance is compared using different training datasets in three popular networks for image classification to check how the augmented data by our SCIT model work as training data. For this experiment, we use the combinations of three datasets as training datasets. Let $T_{o}$ denote the original training data, $T_{t}$ denote the training data by our image translation SCIT method, and $T_{m}$ denote the augmented training data by basic image manipulation. $T_{t}$ individually shows potential with an average accuracy of 79.84 on the testing data, even though its performance is worse than $T_{o}$. One of the reasons is that there are some translation failures in our SCIT model, as shown in Figure 6, which is common with deep learning-based image generation, such as BigGAN (Brock et al., 2018). However, $T_{t}$ coupled with $T_{o},T_{m}$ improves performance averagely by 3.58% over three classification models. Hence, we conclude that our SCIT model can ease downstream applications to be a data augmentation method.

**Table 5:** Verification of SCIT.

**Training data**	**ResNet18**	**DenseNet121**	**MobileNet v2**
$T_{o}$	86.94 ± 0.69	91.21 ± 0.72	85.27 ± 0.47
$T_{t}$	78.24 ± 1.05	81.38 ± 1.06	79.90 ± 1.51
$T_{o}+T_{m}$	90.01 ± 0.42	94.06 ± 0.41	92.37 ± 0.54
$T_{o}+T_{m}+T_{t}$	94.61 ± 0.38	96.48 ± 0.44	96.28 ± 0.32

*We show the performance of three popular classifier models according to differently combined training datasets with averages and standard deviations. $T_{o}$ denotes the original training dataset, $T_{m}$ denotes the augmented training data by basic image manipulation, and $T_{t}$ denotes the training data by our image translation method*.

### 4.3. Image Classification

**Compared algorithms**. To validate our algorithm, we compare it to other image generation methods using GANs, as GANs have good reputations to produce clear images. The following methods are compared:

- *Baseline*. We adopt the image manipulation-based data augmentation with the original training dataset. We argue that our data augmentation is complementary to other data augmentation methods. In other words, taking more advanced data augmentation methods such as mixup (Zhang et al., 2018) and cut and paste (Dwibedi et al., 2017) could be a stronger baseline for our future study.
- *DCGAN* (Radford et al., 2015). DCGAN-based (Pandian et al., 2019) or label-condition GANs (Valerio Giuffrida et al., 2017; Pandian et al., 2019; Bi and Hu, 2020; Abbas et al., 2021) are GAN-based algorithms to do data augmentation in which the generator produces images from random noises or given labels. We adopt the original DCGAN to do data augmentation and to produce a higher resolution, two more upsampling layers and convolution layers are added to the original DCGAN.
- *LeafGAN* (Cap et al., 2020). LeafGAN aims to keep the background, one of the challenges of CycleGAN, and introduces an attention module to distinguish the foreground and background. Performance in the cucumber dataset shows its superiority over than original CycleGAN.
- CycleGAN*. To get a stronger comparison, we updated CycleGAN. CycleGAN* directly reuses the background from the input image with a mask as input. We emphasize that CycleGAN* is the same as the LeafGAN when the mask is given. Simultaneously, our algorithm degrades to CycleGAN* without keeping the style of the input image during the image translation process.

**Quantitive comparisons**. For each method, five independent training processes are performed. Except for the baseline, all methods are utilized to generate images with resolution 256 in width and height. The number of generated images is also the same except for the baseline. Table 6 shows the performances of image classification for tomato leaves with different deep-learning models. From the table, CycleGAN* and our model can significantly improve the classification accuracy. Simultaneously, our data augmentation method achieves the best accuracy and F1 score overall models, which suggests that choosing a good data augmentation method is the way to obtain a better result. In contrast, DCGAN and LeafGAN can not always boost performance. Moreover, each data augmentation method shows the best performance with DenseNet and the worst with ShuffleNet, which suggests that the classification model is also essential and we should choose a better model in our own applications.

**Table 6:** Comparison results to other methods to perform image classification for tomato leaves with multiple deep learning-based models.

	**Training data**	**Accuracy**	**Precision**	**F1 Score**	**Specificity**
VGG11	Baseline	78.72 ± 3.78	98.95 ± 0.12	86.31 ± 5.21	87.42 ± 1.51
	DCGAN	78.34 ± 6.36	98.30 ± 0.78	86.30 ± 4.67	87.46 ± 1.31
	LeafGAN	87.73 ± 2.47	99.06 ± 0.05	94.03 ± 1.37	89.28 ± 0.46
	CycleGAN*	89.04 ± 2.12	99.21 ± 0.33	95.37 ± 1.55	89.81 ± 0.55
	Ours	**94.36** **±2.63**	**99.46** **±0.13**	**96.50** **±2.03**	**90.14** **±0.51**
ResNet18	Baseline	86.75 ± 0.81	99.07 ± 0.43	92.06 ± 0.61	88.80 ± 0.83
	DCGAN	82.52 ± 0.99	98.77 ± 0.29	89.44 ± 0.74	88.17 ± 0.59
	LeafGAN	90.36 ± 0.82	99.27 ± 0.31	94.49 ± 0.56	89.93 ± 0.39
	CycleGAN*	92.68 ± 0.63	99.35 ± 0.12	95.50 ± 0.33	90.33 ± 0.22
	Ours	**94.61** **±0.38**	**99.68** **±0.07**	**96.59** **±0.12**	**90.65** **±0.19**
DenseNet121	Baseline	90.97 ± 0.40	99.59 ± 0.27	94.94 ± 0.27	90.16 ± 0.40
	DCGAN	90.45 ± 1.28	99.63 ± 0.22	94.52 ± 0.74	89.97 ± 0.39
	LeafGAN	92.02 ± 0.75	**99.77** **±0.15**	95.57 ± 0.59	90.37 ± 0.50
	CycleGAN*	96.01 ± 0.32	99.44 ± 0.22	97.14 ± 0.22	90.16 ± 0.62
	Ours	**96.48** **±0.44**	99.61 ± 0.14	**97.87** **±0.40**	**90.76** **±0.31**
MobileNet v2	Baseline	84.37 ± 2.10	99.05 ± 0.35	90.79 ± 1.34	88.35 ± 0.90
	DCGAN	82.80 ± 1.69	98.94 ± 0.37	89.68 ± 1.12	88.33 ± 0.46
	LeafGAN	91.31 ± 0.53	99.31 ± 0.19	95.17 ± 0.30	89.94 ± 0.39
	CycleGAN*	94.14 ± 0.23	**99.44** **±0.39**	96.59 ± 0.77	**90.22** **±0.42**
	Ours	**96.28** **±0.32**	99.36 ± 0.22	**97.53** **±0.30**	90.10 ± 0.52
ShuffleNet v2	Baseline	71.82 ± 0.66	98.98 ± 0.35	84.76 ± 0.31	88.12 ± 0.38
	DCGAN	78.05 ± 1.82	98.80 ± 0.18	86.43 ± 1.10	87.47 ± 0.46
	LeafGAN	77.84 ± 2.26	**99.15** **±0.31**	88.89 ± 1.45	88.97 ± 0.70
	CycleGAN*	79.56 ± 0.42	99.12 ± 0.11	89.54 ± 0.48	**89.92** **±0.17**
	Ours	**87.44** **±0.69**	**99.15** **±0.46**	**92.43** **±0.87**	89.76 ± 0.69

*The boldfaces show the best average evaluation metric for each classification model*.

**Figure 7:**
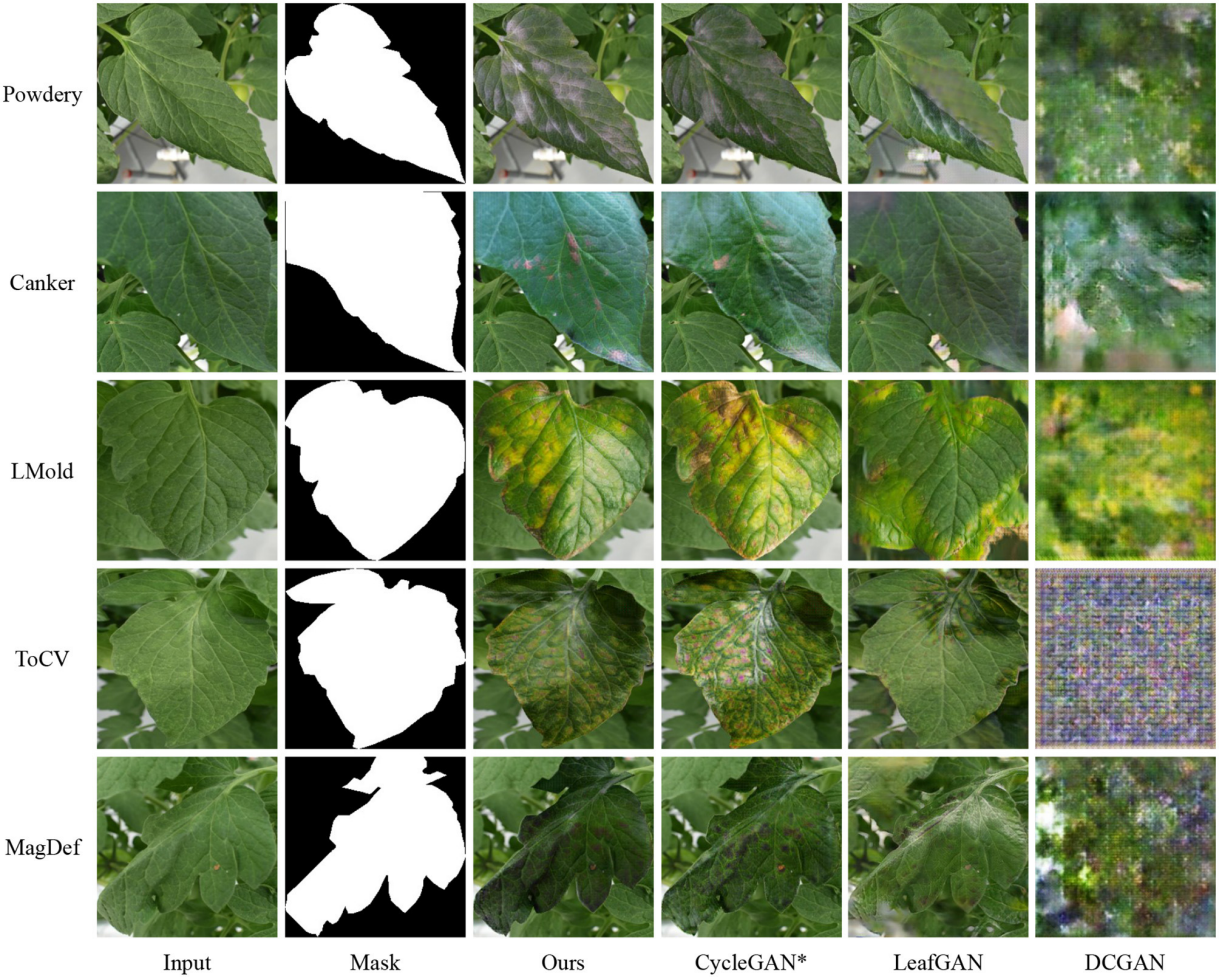
Qualitative results of different algorithms to do image translation for image classification.

**Qualitative results**. Figure 7 shows several generated samples from each algorithm. First, DCGAN can learn similar patterns such as the white part for the powdery and yellow part for LMold, but produces poor images and, in ToCV, fails. The visual results verify its impact in Table 6. Moreover, LeafGAN gives plausible images but its attention module is hard to find decent objects to be translated. Hence, it tends to change the background and fails to do image translation such as the canker image. In contrast, CycleGAN*, an advanced LeafGAN with a perfect attention module, achieves much better results and hence boosts the classification performances. Furthermore, our method, adopting a style loss to maintain the style during the image translation and hence taking the variations from the source domain to the target domain, obtains decent visual images.

### 4.4. Object Detection and Instance Segmentation

As discussed before, our algorithm reuses the annotation for object detection and instance segmentation. As DCGAN is not image translation-based and LeafGAN can not maintain the annotations, we compare our algorithm to CycleGAN* in this subsection. To do object detection and instance segmentation, mmDetection^5^ is borrowed as it supports many models. For object detection, we leveraged FasterRCNN, MaskRCNN, and PointRend. In a different paradigm, YOLO aiming to achieve high-speed object detection is also used. Except for object detection, MaskRCNN and PointRend are deployed to do instance segmentation. As λ_*sty*_ = 2 shows its superiority in classification, we use it as the default value and compare it to baseline and CycleGAN*. For each model, we also execute it five times separately and compute the average performance.

**Figure 8:**
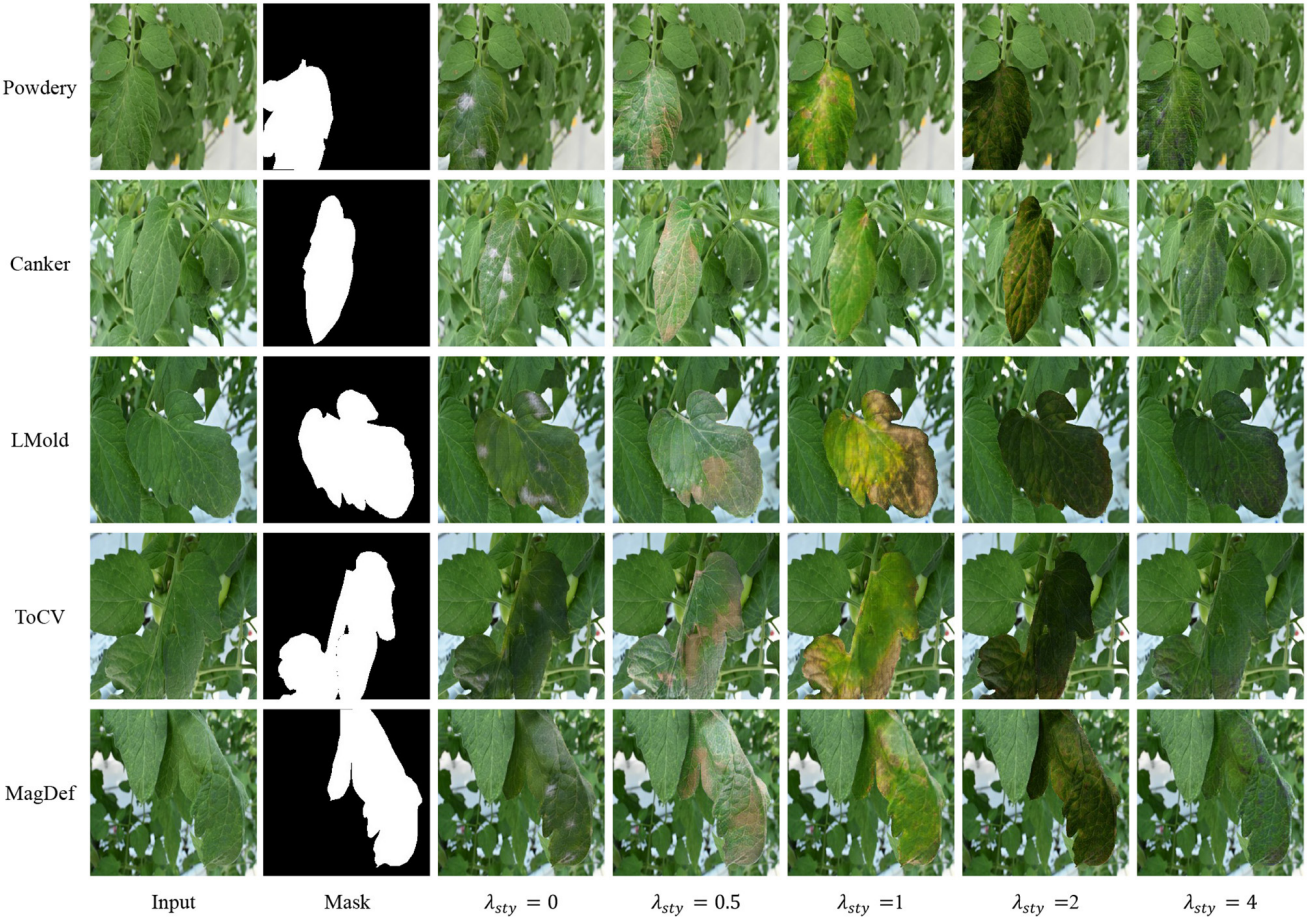
Some generated samples to do data augmentation for object detection and instance segmentation in which one image could include more than one leaves but we can just translate the desired one and maintain the others.

**Figure 9:**
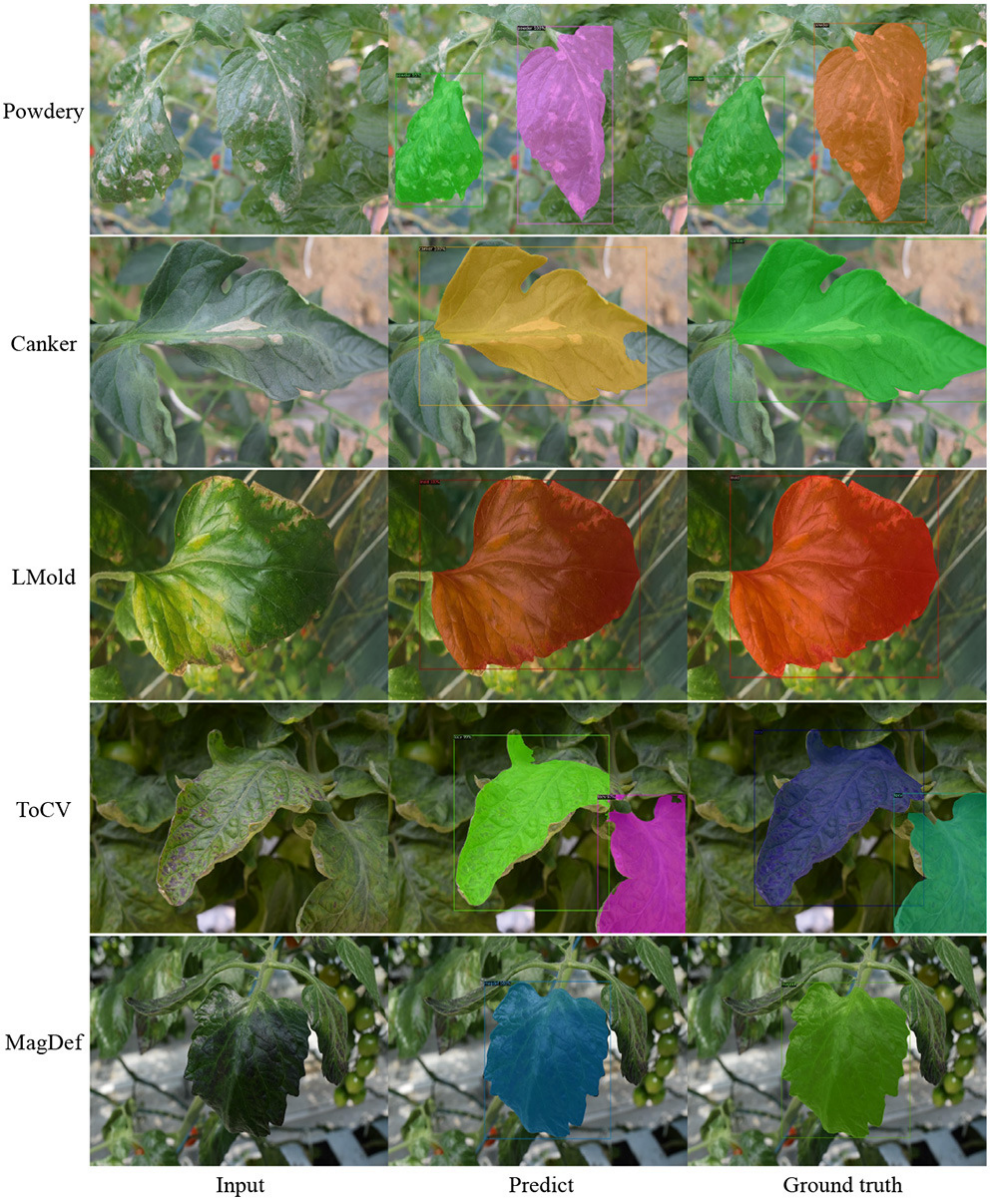
Result samples of instance segmentation in different diseases with our SCIT model as data augmentation model and λ_*sty*_ = 2. The predicted results are decent compared to the ground truth. Zoom in to see the bounding boxes.

Table 7 displays the comparison. We observe that while the CycleGAN* improves the performance, our method gives more improvements. Except for AP50 in YOLO-v3, our model achieves the best mAP and AP50. Besides, we find that FasterRCNN, MaskRCNN, and PointRend obtain similar results and better results than YOLO-v3 to do object detection. Figure 8 displays some generated samples as data augmentation for object detection and instance segmentation, in which one image includes more than one healthy leaf but we choose one of them to do data augmentation with necessary annotations as output. Figure 9 illustrates several samples of instance segmentation results using our SCIT model as a data augmentation method. As shown in the figure, the predicted results are highly competent to the ground truth.

**Table 7:** Performance of object detection and instance segmentation for tomato leaves in multiple deep learning-based models.

	**Baseline**		CycleGAN*		**Ours**	
	**mAP**	**AP50**	**mAP**	**AP50**	**mAP**	**AP50**
FasterRCNN	49.5 ± 0.007	76.8 ± 0.010	50.7 ± 0.015	77.2 ± 0.012	51.5 ± 0.019	77.4 ± 0.015
MaskRCNN	52.4 ± 0.014	79.6 ± 0.015	55.6 ± 0.010	80.4 ± 0.011	56.6 ± 0.005	80.5 ± 0.007
PointRend	51.7 ± 0.009	79.4 ± 0.006	52.8 ± 0.007	80.9 ± 0.011	53.4 ± 0.007	81.1 ± 0.011
YOLO-v3	29.5 ± 0.007	58.7 ± 0.013	31.2 ± 0.015	63.2 ± 0.025	32.6 ± 0.013	65.6 ± 0.024
MaskRCNN	62.6 ± 0.015	80.1 ± 0.009	66.6 ± 0.006	80.0 ± 0.012	67.1 ± 0.010	79.9 ± 0.007
PointRend	56.1 ± 0.023	80.6 ± 0.007	67.6 ± 0.007	81.0 ± 0.010	68.3 ± 0.006	81.3 ± 0.008

*Four models have been used to do object detection, while two to do instance segmentation. Red font is utilized to show the best average mAP for each model while blue is adopted to show the best average AP50. In general, higher mAP and higher AP50 are better*.

## 5. Conclusion and Future Work

In this study, we introduced a new data augmentation method to improve the abnormality recognition for tomato leaves, termed SCIT which aims to keep the image style from the source image when doing image translation. Armed with this data augmentation paradigm, the data variation in the variation-minority classes is enlarged by the variation-majority class. Simultaneously, we extended the data augmentation from image classification to object detection and instance segmentation which is more competing to do downstream applications. Experimental results validated that the proposed data augmentation method outperforms the baseline and popular methods, in image classification, object detection, and instance segmentation. Although our algorithm was verified to be useful, our future study includes how to integrate different types of data augmentation methods to facilitate the data-hungry deep learning methods, such as advanced image manipulations (mixup Zhang et al., 2018 and cut and paste Dwibedi et al., 2017). We hope that our study can stimulate the community to use a more powerful data augmentation method to improve the recognition performance for diseases or other abnormalities in the agricultural field where data are hard or expensive to collect.

## Data Availability Statement

The original contributions presented in the study are included in the article/supplementary material, further inquiries can be directed to the corresponding authors.

## Author Contributions

MX conceived the idea and designed the algorithm, conducted the experiments, and wrote the manuscript. SY supervised the project, analyzed the algorithm, and contributed to part of the writing and overall improvement of the manuscript. AF collected the original images and performed the preliminary experiment on object detection and revised the manuscript. JY enriched the idea, contributed to data annotation, and improved the manuscript. DP conceptualized the paper, supervised the project, and got funding. All authors read and approved the manuscript.

## Funding

This research was supported by the Basic Science Research Program through the National Research Foundation of Korea (NRF) funded by the Ministry of Education (No. 2019R1A6A1A09031717); by the National Research Foundation of Korea (NRF) grant funded by the Korea government (MSIT) (NRF-2021R1A2C1012174); and by Korea Institute of Planning and Evaluation for Technology in Food, Agriculture and Forestry (IPET) and Korea Smart Farm R&D Foundation (KosFarm) through Smart Farm Innovation Technology Development Program, funded by Ministry of Agriculture, Food and Rural Affairs (MAFRA) and Ministry of Science and ICT (MSIT), Rural Development Administration (RDA) (421027-04).

## Conflict of Interest

The authors declare that the research was conducted in the absence of any commercial or financial relationships that could be construed as a potential conflict of interest.

## Publisher's Note

All claims expressed in this article are solely those of the authors and do not necessarily represent those of their affiliated organizations, or those of the publisher, the editors and the reviewers. Any product that may be evaluated in this article, or claim that may be made by its manufacturer, is not guaranteed or endorsed by the publisher.

## Footnotes

^1^ https://github.com/xml94/SCIT

^2^ https://pytorch.org/vision/stable/models.html#torchvision.models.vgg19

^3^ https://github.com/mseitzer/pytorch-fid

^4^ https://github.com/bearpaw/pytorch-classification

^5^ https://github.com/open-mmlab/mmdetection
